# Evaluation of the Presence and Viability of *Mycobacterium bovis* in Wild Boar Meat and Meat-Based Preparations

**DOI:** 10.3390/foods10102410

**Published:** 2021-10-12

**Authors:** Maria T. Clausi, Lucia Ciambrone, Mariagrazia Zanoni, Nicola Costanzo, Maria Pacciarini, Francesco Casalinuovo

**Affiliations:** 1 Catanzaro Section, Experimental Zooprophylactic Institute of Southern Italy (IZSM), 88100 Catanzaro, Italy; lucia.ciambrone@cert.izsmportici.it (L.C.); francesco.casalinuovo@cert.izsmportici.it (F.C.); 2 National Reference Centre for Bovine Tuberculosis by *Mycobacterium bovis*, Experimental Zooprophylactic Institute of Lombardia and Emilia Romagna (IZSLER), 25124 Brescia, Italy; mariagrazia.zanoni@izsler.it (M.Z.); maria.pacciarini@izsler.it (M.P.); 3 Department of Health Science, Catanzaro University “Magna Græcia”, 88100 Catanzaro, Italy; costanzo.nic@unicz.it

**Keywords:** wild boar, *Mycobacterium bovis*, tuberculosis, meat, zoonosis

## Abstract

The aim of the present study is to provide information about the ability of *Mycobacterium bovis* to survive within wild boar (*Sus scrofae*) meat and meat-based preparations and the duration of this survival, and to consider the preservation of its infectious potential toward humans and animals. Meat samples were artificially contaminated with an *M. bovis* field strain and then stored at −20 °C, while two sausages batches were contaminated with the same field strain at two different concentrations, 10^5^ CFU/g and 10^3^ CFU/g, before storing them in proper conditions to allow for their ripening. A third sausage batch was contaminated by adding 2 g of wild boar lymph nodal tissue with active tuberculous lesions to the meat mixture. Bacteriological and biomolecular (PCR) methods were used to test the meat and sausage samples every 60 days and every 7–10 days, respectively. *M. bovis* was detected as still alive and viable on the frozen meat for the last test on the 342nd day, while from the sausage samples, *M. bovis* was isolated until 23 days after contamination. Our results indicate that *M. bovis* can stay alive and be viable for 23 days within sausages prepared with contaminated meat from infected wild boars. These products are usually eaten as fresh food after grilling, often cooking at a temperature that does not ensure complete inactivation of the pathogenic microorganisms present, which can pose a risk for humans to develop zoonotic tuberculosis.

## 1. Introduction

Currently, bovine tuberculosis is still an important global issue for human health and causes severe economic impact due to the difficult eradication process of the disease in cattle farms and due to the spread of this infection to wildlife. Indeed, as occurs in multi-host epidemics, tuberculosis control and eradication in farmed hosts cannot be reached if it is not carried out together with disease control on wild reservoirs. Actually, few options for tuberculosis control in wildlife are available [1]. An important preventive action must be carried out by consumers and operators working in slaughterhouses to highlight the risk associated with the consumption of raw or undercooked meat [1].

A recent survey, conducted among Italian wild mammals hunters about the use of hunted animal meat, reports that 32% of them consume all meat within their family, 28% give some meat to friends and relatives, 3% give all meat away, and 17% give away about 1/3–1/2 of the meat, while 20% of the interviewed hunters did not answer the question [2]. We can therefore deduce that game meat consumption is no longer limited to hunters’ families but involves a wider range of consumers too, up to distributing game products within group catering.

In recent years, the game meat sector has shown a steady increase in demand and supply thanks to the significant increase in the number of some wild species, especially some ungulates such as wild boar (*Sus scrofae*). The growing commercial interest toward the possibility of wild boar meat as food has nevertheless also highlight health hazards linked to many viral, bacterial, and parasitic pathogens that this animal species can spread to other animals, both wild and domestic ones, and to humans, as a result of direct and/or indirect contact with animals or by ingesting their meat [3,4]. Tuberculosis caused by *Mycobacterium bovis* (*M. bovis*) is a widespread infection in the wild boar population of many European countries [5], including Italy, where bovine tuberculosis is still present within southern regions despite sanitation plans started about 40 years ago. Wild boars infected by *M. bovis* can become direct or indirect sources of contagions for humans and other wild and domestic animals [6,7]. In this wild ungulate, infection affects many lymph nodes and organs, mainly those belonging to the respiratory and digestive tracts, producing anatomopathological pathognomonic lesions on retro-pharyngeal, sub-mandibular, bronchial, mediastinal, hepatic, mammary, sub-iliac, and popliteal lymph nodes and on the lungs and liver, even though we cannot exclude affections of other areas and organs.

With regard to food safety, at present, few studies concerning the risk of transmission of *M. bovis* to humans due to the consumption of wild boar meat exist despite the worrying prevalence of infections found in this species within many Italian areas [8,9,10]. This hazard is often overlooked because of the cooking process under which meat usually undergoes [11,12,13]. However, recent studies confirmed *M. bovis* contamination in carcasses of regularly slaughtered cattle [14,15] and frozen carcasses of regularly slaughtered buffalo [16] ready for commercial distribution. In light of the above-reported observations for cattle and buffalo meat, admitting to a potential risk of infection by *M. bovis* for humans due to the consumption of wild boar meat seems reasonable [17]. This likely hypothesis should not be overlooked, considering that the infectious load needed to cause human or animal disease through food-borne routes is still unknown.

In Italy, sausages made from wild boar meat are food products in high demand and are often consumed within public catering and during gastronomic festivals. The risk of zoonotic tuberculosis linked to the consumption of these foodstuffs can be due to inadequate cooking if the sausages were produced using meat from wild boars infected by *M. bovis* or meat contaminated during slaughtering activities involving groups of animals. At least three cases of human tuberculosis caused by *M. bovis* in poachers and/or their relatives who had eaten wild boar meat were observed in the geographic area involved in this study. These data have been derived from local findings of some human health structures, but they have not been published as a scientific paper/report.

The need for a risk assessment of human tuberculosis infection with *M. bovis* as a result of eating wild boar fresh and processed meat (e.g., sausages) is well justified by the worrying prevalence of tuberculosis infections observed in wild boar populations as well as by the increase in human cases of the disease noticed within our territory, mainly observed among butchers, hunters, and common consumers (unpublished personal data).

Our study intends to produce data about human infection hazards related to consuming meat and cured meats produced using wild boar meat contaminated with *M. bovis*. For this purpose, two main evaluations were carried out:

- A risk evaluation for *M. bovis* contamination of carcasses of wild boar;
- A risk evaluation for *M. bovis* contamination of meat and sausages produced using wild boar meat.

The mycobacterium involved was detected alive and viable until 23 days after the preparation of artificially contaminated sausages and until 342 days on frozen meat samples.

## 2. Materials and Methods

### 2.1. M. bovis Contamination of Carcasses of Wild Boar

We considered a group of 18 animals killed during a single hunting trip. During veterinary post-mortem inspection, 3 out of 18 killed wild boars showed disseminated tuberculous lesions in different organs and were to be destroyed. The remaining 15 carcasses were declared suitable for human consumption and cold stored until further meat processing.

Sterile swabs were used for microbiological sampling from the surfaces of the muscle tissue of these 15 carcasses; all samples were tested for *M. bovis* presence using bacteriological examination (BE) and polymerase chain reaction (PCR).

### 2.2. M. bovis Contamination of Meat and Sausages Produced Using Wild Boar Meat

In order to assess the risk linked to consuming wild boar meat and sausages, we needed to simulate the production and experimental contamination of these foodstuffs. For our purposes, we planned and performed different activities carried out during four experimental sessions:

- During the first session, we prepared fresh sausages from minced wild boar meat, and experimental contamination of the mixture was achieved by adding infected material consisting of small portions of lymph nodal tissue with active tuberculous lesions (the first experimental batch). The mixture, weighing about 1200 g, was made up of wild boar meat that was minced and seasoned with salt and ground pepper. We contaminated it by adding about 2 g of pharyngeal lymph nodes from a wild boar with clear active tuberculous lesions and which was previously tested positive for the presence of *M. bovis* through bacteriological examination. Fresh sausages, weighing about 100 g, were produced using the contaminated mixture, and microbiological evaluations were performed on six of these portions with 7–10 day intervals. The sausage samples were stored at room temperature (10–18 °C) in a controlled environment for 37–43 days. During this storage period, the fresh product underwent a ripening process, which allowed the sausages to dry out and to experience all of the biochemical modifications that characterize aged, cured meat;
- During both the second and the third sessions, we produced fresh sausages using the same procedure previously described: six of them were used for experimental tests and were stored at the same conditions already described. For experimental contamination of these batches, we used a field strain of *M. bovis*, which was cultured and added to the matrices at different concentration levels: 10^5^ CFU/g for the second experimental batch and 10^3^ CFU/g for the third experimental batch;
- During the last session (the fourth experimental batch), 700 g of wild boar loin meat was cut into slices of about 100 g in weight, and the surfaces of each slice were contaminated using a sterile swab soaked with a suspension of 10^3^ CFU/mL of the same field strain of *M. bovis* used for the previous two sessions. Contaminated slices of meat were stored at −20 °C. Single portions of this meat were tested with BE and PCR, with intervals of about 60 days.

The strain of *M. bovis* used to contaminate the experimental samples was the SB120/4,5,5,3,3,10,4,4,4,3,6,5 genotype that was isolated from bronchial lymph nodes belonging to a wild boar killed during a hunting trip and that, during veterinary post-mortem inspection, presented a complete primary bronchopulmonary tubercular complex. This strain was chosen from a collection of 14 different strains, with all of them isolated from wild boars, because it represents the most common genotype in Italy, and it was often detected within the wild boar population in Calabria starting in 2008. Over the years, many epidemiological data have highlighted that this strain infects both cattle and wild boars in the same area. The genetic profile of this strain was identified using the spoligotyping method together with an ETR (exact tandem repeat) loci analysis in order to reveal any possible homology with other circulating strains within the same territory [18,19,20,21,22,23]. The order of the employed markers is reported in Table 1.

### 2.3. Analytical Procedures

All experiments were performed in a Biosafety level 3 laboratory according to standard procedures intended for handling tubercular mycobacteria. Sausages produced as described above for first, second, and third experimental batches, and later put in a controlled environment at 10–18 °C, underwent BE and PCR tests throughout the survey period according to the following procedures.

- Bacteriological examination: we used a method employing a combined system of liquid and solid media, but the latter medium was used only for samples that tested positive on the liquid medium. An amount of 10 g of meat/sausage was previously minced and then diluted to 1:2 using an 8.5% saline solution, before being homogenized and decontaminated using a 4% sodium hydroxide solution; the sample thus processed was incubated at 37 °C ± 1 °C for 30 min. After this phase, a 10% sulfuric acid solution was added to the homogenized sample in order to neutralize its alkalinity, and phenol red was used as a pH indicator. The sample was then centrifuged at 3000 rpm at 20 °C for 15 min, the supernatant was discharged, and the pellet was re-suspended with 2 mL of a phosphate buffer at pH 6.8. For the first culturing step on the liquid medium, a Mycobacteria Growth Indicator Tube (MGIT^TM^) was used, and an aliquot of 0.5 mL of the phosphate buffer suspension was inoculated on MGIT^TM^ 960, previously added with 0.8 mL of PANTA MGIT^TM^, made up of a growth supplement and a mix of antibacterial drugs. The vials were then incubated at 37 °C in the specific incubator Bactec^TM^ MGIT^TM^ 960 System (Becton Dickinson); the instrument automatically identifies and marks positive vials if mycobacterial growth occurs. From each positive vial, 0.2 mL of the sediment was transferred to solid egg-based media, Lowenstein-Jensen (LJ) and Stonebrink (ST); it was further incubated at 37 °C ± 1 °C for a maximum period of 8 weeks, and was monitored weekly to verify the growth of typical colonies. If any typical colony was not observed at the end of this last incubation period, the sample was reported as negative. All bacterial isolates belonging to *Mycobacterium* spp. were sent to the National Reference Centre for Bovine Tuberculosis at the Experimental Zooprophylactic Institute of Lombardia and Emilia Romagna in order to perform further evaluations about their genetic profiles;
- Polymerase chain reaction: DNA extraction was performed by applying mechanical lysis to the sample tissue, and the supernatant was then collected and processed using a commercial extraction kit (QIAamp DNA mini kit, Qiagen, Germany). Extracted DNA underwent a real-time PCR targeting the specific genetic insertion region IS6110, a highly repeated sequence in strains belonging to the genus Mycobacterium, using primers complementary to the target sequence and that amplify a 209 bp amplicon. Any possible presence within the sample of substances able to inhibit the reaction was monitored by adding an internal control to the PCR mix; the primers and probe used are presented in Table 2.

For the amplification procedure, the commercial kit Quantifast pathogen +IC KIT (Qiagen) was used, and the following PCR protocol was adopted: initial denaturation at 95 °C for 5 min, then 45 cycles of denaturation at 95 °C for 15 min and annealing/extension at 60 °C for 30 min. Samples were reported as positive if they showed the following cycle threshold (CT) value for FAM: 5 ≤ CT ≤ 38. The whole process was previously validated as an internal method and showed values ≥95% for both sensitivity and specificity.

Each sausage portion used as an analytical sample was checked starting from day 0 (T_0_, preparation date) and about every 10 days for a total of six examinations performed with each method, aiming to detect the presence and viability of inoculated *M. bovis* by means of PCR tests, which pointed out the presence of deoxyribonucleic acid (DNA), and of isolation through cultural techniques, making marking its viability and fertility possible. Meat portions from samples from the fourth batch were similarly tested following the different time intervals reported in the previous sub-paragraph.

Neither the BE nor PCR methodologies were used here to quantify the level of contamination but, rather, to simply reveal the presence of the mycobacteria, so no colony counting was performed after the cultures, and no calibration curve was created for the molecular analysis.

### 2.4. Statistical Analysis

The experimental results underwent statistical analysis using SPSS^®^ (Statistical Package for Social Science), an IBM^®^ (International Business Machines Corporation, Armonk, NY, USA) software. The tests employed for this purpose were chosen by taking into account the limited amount of sample units and the nature of the binary outcome produced by the laboratory analyses, which have to be processed as a categorical/nominal variable.

In the beginning, curves following the Kaplan-Meier method were generated, and we evaluated them in a “time to failure” model, referring to the failure to detect/isolate the pathogen as an event of interest for both analytical methods; indeed, the data present analytical detection more than the presence of live mycobacteria. Then, we used a logarithmic rank test (the Cox-Mantel log-rank test) in order to obtain a first evaluation of the differences between groups of data. To confirm the significance of the results and to identify the effect of each considered variable within the system, we also performed a regression according to the Cox model after verifying that it was adequately applicable. We considered *p* values equal to or below 0.05 as being statistically significant, and hazard ratios (HRs) with a 95% confidence interval (CI) were calculated. We examined the ability of the two analytical methods used to detect *M. bovis* contamination and the effects of other parameters, such as different contamination levels, types of matrices, and the contamination procedure, considering all of the analytical results from the first three batches (whole data set) and the separate results obtained from the two analytical methods. For each considered variable parameter, we calculated the HR by comparing the outcomes produced by data groups characterized by differences related to that specific parameter, and for this purpose we needed to choose a reference/control group. Within each data set analysis, the chosen category was the one that produced a median value, referred to as the period of time during which detection of the pathogen in question was possible, lower than those observed for the other groups.

Due to the different time intervals adopted for the tests on samples from the fourth experimental batch compared with those adopted for the tests on the other batches, the data originating from these analyses were excluded from the above-mentioned comparisons. Moreover, with regard to contamination levels, on the basis of the limited available bibliography and of the results from our previous studies, we hypothesized that contamination realized by adding naturally infected lymph nodes can be considered and used to produce contamination at intermediate levels if compared with the levels achieved on the second and the third experimental batches.

## 3. Results

### 3.1. Evaluation of M. bovis Contamination of Carcasses of Wild Boar

For wild boar carcasses cold stored and sampled by swabbing the muscle surfaces, PCR tested positive for the presence of *M. bovis* for 2 out of the 15 animals. The BE results, instead, were always negative.

### 3.2. Evaluation of M. bovis Contamination of Sausages Produced Using Wild Boar Meat (First Experimental Batch)

Analyses were performed at intervals of about 7–10 days starting from T_0_. As presented in Table 3, for the first three checks (T_0_, T_1_, and T_2_), *M. bovis* was always detected in the samples by PCR while BE always tested negative. At the fourth check carried out 23 days after preparation/contamination date (T_3_), *M. bovis* was detected by both PCR and BE. After further analysis, the isolated strain proved to be genetically correlated with the strain obtained from organs of the infected animal used as a source of the retro-pharyngeal lymph nodes added to the experimental samples. At the fifth and the sixth checks (T_4_ and T_5_), PCR still tested positive, but no bacterial growth was found using BE.

### 3.3. Evaluation of M. bovis Contamination of Sausages Produced Using Wild Boar Meat (Second Experimental Batch)

For the second batch, samples were checked at intervals of about 7–10 days starting from T_0_, and the results are shown in Table 4. For the first four checks, *M. bovis* was always detected in the matrices by PCR, and BE tested positive until 22 days after preparation/contamination (T_3_). At T_4_ and T_5_, instead, *M. bovis* was detected by PCR while BE tested negative. The isolated strain was proven to be genetically correlated with the strain used for the experimental contamination.

### 3.4. Evaluation of M. bovis Contamination of Sausages Produced Using Wild Boar Meat (Third Experimental Batch)

The results of the third batch are shown in Table 5. For all checks, BE tested negative for the presence of *M. bovis* while PCR detected its presence at T_0_, T_1_, and T_4_.

### 3.5. Evaluation of M. bovis Contamination of Wild Boar Meat (Fourth Experimental Batch)

*M. bovis* was always detected by both PCR and BE tests carried out at time intervals of about 60 days for the fourth batch (Table 6). The check scheduled at T_2_ was not performed due to a sanitary emergency involving SARS-CoV2.

### 3.6. Statistical Analysis

**Figure 1:**
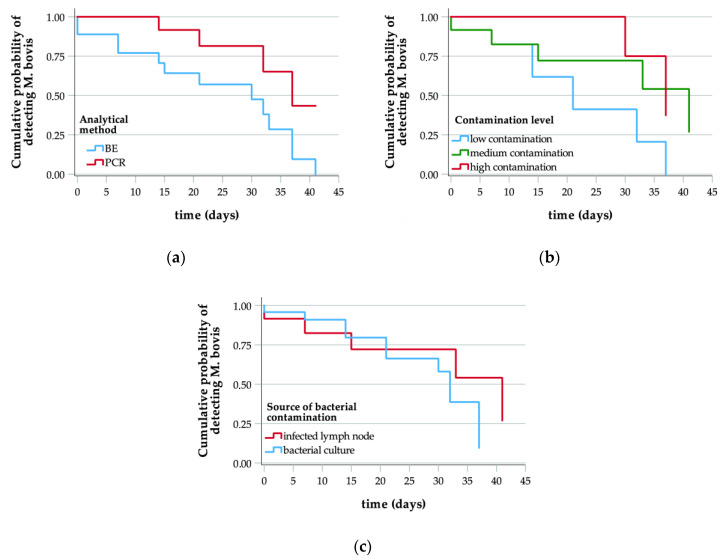
Curves following from the Kaplan-Meier method concerning the whole data set from first, second, third experimental batches. The panels present the data compared for (**a**) the analytical method, (**b**) the contamination level of the matrix, and (**c**) the contamination source/procedure.

Data from the fourth experimental batch were only analyzed through the production of Kaplan-Meier curves related to the two analytical methods; we could not perform further evaluations due to the two groups of data fully overlapping. Indeed, both techniques enabled the detection of contamination during the whole observation period (except for tests at T_2_ that were not carried out). On the other hand, Kaplan-Meier curves related to data from the other batches indicate that PCR can detect the pathogen for a longer period than BE (mean values of 37 days and 30 days, respectively, are provided for the whole data set) (Figure 1a).

With regard to this parameter, we observed differences in the cumulative probability of detecting the pathogen at 31 days ranging from 28%, concerning contamination by bacterial cultures (more specifically 17% and 50% referred, respectively to the third and second batches), to 50% when samples were contaminated using naturally infected tissue (first batch).

Figure 1b,c present the curves obtained from data separated according to sample contamination level and to the contamination source/procedure, respectively. Concerning the first parameter, the median time value during which detection was achieved was lower for samples from the third batch (12 days), and for both methods the values observed were higher for samples from the second batch. Separately examining the data produced by cultural and molecular analyses, dissimilar images appeared from a comparison of the curves related to the different contamination levels. Namely, for BE the first and the third batches generated similar median values, while the results referred to the second batch clearly differ, proving to be higher than the previous ones. On the other hand, the PCR data produced a curve related to the first batch that showed a median value quite similar to that from the curve of the second batch, and we can point out a lower value only for the batch characterized by a low contamination level. Concerning the second parameter, the median time values were broadly similar, despite a slightly lower value for samples contaminated by adding infected lymph nodes, but by evaluating the data separately according to the analytical technique used, we noticed some differences. All curves produced with the BE results have a similar trend, and detection was possible for a slightly longer period from samples of the group made up from the second and third batches. Instead, for the PCR curves the trend diverged and, although periods enabling pathogen detection were similar to what was observed for BE with regard to the two batches contaminated with bacterial cultures, this period was extended for samples from the first batch and reached the end of the observation period.

Table 7 presents the main results of further statistical data processing, together with reference categories used to calculate HR values. The choice to adopt a multivariable regression model allowed us to significantly improve the likelihood values when compared with the simple regression models.

The difference between the results of the two methods used to reveal contamination throughout the observation period was proven to be statistically significant both in the log-rank test and Cox regression (Table 7), but by separately analyzing each single batch, the above-mentioned difference maintained significance only within the first experimental batch (*p* = 0.025). For this parameter, we calculated a general HR value of 0.308, and values lower than 1 were observed in relation to the single batch analyses, too (the maximum HR value was related to the third batch: HR = 0.667). Additionally, the different contamination degrees were proven to affect the analytical results significantly, and an HR value of 0.459 for the whole data set was observed. Hazard ratio values < 1 were calculated in relation to each method, too, even if a significant difference between data groups was confirmed only for the PCR analyses; however, for these latter data, we observed a loss of significance when the multivariable model was adopted (see Table 7). With regard to the same variable, a paired comparison performed within the entire data set enabled us to point out statistically significant differences only between data from the second and third batches (*p* = 0.015), and the data were confirmed using a multivariable analysis too (*p* = 0.045). A comparison between the results from the first and third batches produced *p* values a little above the set significance threshold (*p* = 0.066), and for this comparison, we observed the only significant data that was revealed by separating the analytical results by the method employed (*p* = 0.046 at log-rank test for PCR results), even though significance was not confirmed when adopting the Cox model. Finally, concerning the effects of different contamination sources or food matrices, as presented in Table 7, the statistical tests showed the absence of a significant difference between the two groups. Related HRs were different depending on the data set from which they originated, but all three data processing procedures produced higher HR values as a result of the multivariable analysis compared with the results obtained from a simple regression model (see Table 7). From the overall comparison and with regard to the molecular tests, the multivariable regression produced HR values < 1; conversely, for the bacteriological exams we observed an HR value that was slightly lower than 1 from the simple regression analysis but slightly higher than 1 from the multivariable one.

## 4. Discussion and Conclusions

The present study produced data and useful information about the ability of *M. bovis* to survive viably within matrices such as wild boar meat and sausages. The presence of tuberculosis by *M. bovis* in wild boar is well documented, and it allows the wild reservoir to infect cattle farmed within the same area, affecting the outcome of the eradication programs. This wild species can often move across territories, which can result in a re-introduction of the disease within cattle farms that had previously become tuberculosis-free. The risk of zoonotic tubercular infection in humans is then linked both to the persistence of tubercular infection in cattle and to its presence in a wild boar population.

The possibility of human contagions from the meat of slaughtered animals is one of the epidemiological issues related to zoonotic tuberculosis by *M. bovis* that still needs to be definitively verified. Many international authorities operating in the field of food safety, among which include the European Food Safety Authority (EFSA) and the Advisory Committee on the Microbiological Safety of Food (ACMSF), developed and provided opinions based on scientific findings. They report the outcome that, despite the possibility of bovine meat having a role in spreading *M. bovis* to humans, the related risk is substantially low [25] or even absent [26], and that it can be ignored with a medium uncertainty level [27,28] due to the cooking process that meat undergoes before consumption and thanks to the control measures enforced. Nevertheless, we need to highlight that the same documents do not exclude the possibility that meat, in specific situations, can be the origin of cross-contamination of other foodstuffs, utensils, work surfaces, refrigerators, and kitchens. The same bovine meat, if coming from facilities also slaughtering cattle that test positive in the Mantoux test, can turn out to be contaminated by *M. bovis* when it enters a commercial market [14,15]. We cannot even exclude that consuming undercooked or uncooked meat can occur considering the recently reported dietary habits.

The first experimental batch was arranged in order to reproduce a similar situation to one that might occur under natural conditions during traditional practices for wild boar sausage preparation by hunters’ families. Indeed, during these procedures, fragments of organs or lymph nodes affected by tuberculous lesions can enter the mixture used to produce processed meat. In wild boars with active tuberculosis, the sub-iliac, popliteal, and mammary lymph nodes that are situated near muscles usually used in cured meat production are often affected. The results of our experimental tests indicate that *M. bovis* can stay alive and viable within sausages up to 23 days after the preparation date (T_3_). Actually, the BE-negative results observed at T_1_ and T_2_ can be due to an uneven distribution of the contaminating material in the meat mixture. Indeed, as with the other members of the MTBC (Mycobacterium tuberculosis complex), *M. bovis* is an endocellular pathogen that survives and replicates mainly within macrophages, whereas it does not replicate in the external environment or at conditions similar to those adopted for our experimental trials [29].

Similar results were obtained also using 10^5^ CFU/g to contaminate samples of the second experimental batch: the re-isolated strain of *M. bovis* SB0120 was alive and viable up to 22 days after batch preparation. This strain of *M. bovis* is the most widespread genetic profile in Italy and has also been isolated in four different cattle farms within the area evaluated in our study.

Analyses regarding the third experimental batch that involved the use of 10^3^ CFU/g to contaminate the samples did not the enable re-isolation of *M. bovis* from any of the tested samples. The low concentration of infecting materials dispersed in the matrix was probably lower than the recovery limit of the employed cultural method, as also demonstrated by the PCR test being positive up to 32 days (T_4_). However, we cannot exclude the possibility that low bacterial levels, insufficient to induce growth on the cultural media, were instead able to produce disease onset in humans and animals. With regard to the latter, in fact, the fact that 1 CFU of *M. bovis*, containing six to ten viable bacteria, via endo-tracheal inoculation is a sufficient amount to cause disease in cattle and badger (*Meles meles*) has been proved by experimental studies [30,31].

Within the fourth batch, we always detected *M. bovis* as being alive and viable in experimentally contaminated meat stored at −20 °C, even 342 days after contamination. Although freezing at −80 °C ensures an improvement in obtaining live and viable mycobacteria [32], we chose to freeze samples at −20 °C because this is the procedure adopted by hunters’ families and within public catering to store wild boar meat supplies. Operations involving skinning, evisceration, cutting, and storing the meat of animals killed by hunters themselves, often in poor hygienic conditions, produce a concrete risk for meat contaminated with pathogenic bacteria, including *M. bovis* [33].

For the statistical analysis, the data obtained provide interesting ideas and leads to consistent conclusions, despite the lack of statistical significance shown by some comparison and the limitations due to the 95% CI ranges. Indeed, the confidence interval ranges related to data from the molecular analyses are wide, and this condition limits the reliability of the calculated HR values; an improvement in the strength of this index could be achieved by increasing the sample size on further studies.

In general, the results produced by our experiments showed a reduction of 69% in the probability of not detecting contamination in the tested samples if a molecular technique was adopted rather than a microbiological one, with a statistically significant impact on the analytical results; the extent of reduction was obviously more limited when low contamination levels were involved. A temporal analysis of the data equally indicated that BE provides a relatively suitable performance similar to that shown by PCR (cumulative detection rate ≥ 75%) until the fourth week only if a matrix was contaminated with high bacterial counts, a condition that could affect the number of surviving mycobacteria during the storage period of foodstuffs. Instead, when low contamination occurred, an efficiency loss was already observed from the third week for PCR too. Of course, with the increase in the degree of contamination, both methods showed an improvement in their ability to detect contamination, and, in general, the risk of not detect the mycobacterium gradually and significantly decreased (*p* < 0.05 for both the paired comparison between the second and third batches and the overall Cox model generated from the whole data set). However, we have to highlight that cultural analyses underwent a more limited reduction in the risk of testing negative, while for PCR this reduction was greater and occurred earlier. With regard to the last considered variable, even if we generally noticed that the detection of the pathogen seemed to be slightly easier or more probable throughout the observation period in the batch contaminated with a naturally infected material and that a lack of significance in the difference between data groups separated on the basis of the contamination source emerged, our remarks are probably the result of the different performance from PCR with respect to BE and of the different requirements underlying the two methods. In fact, the addition of cultured bacterial colonies to the food matrix ensured, at least in the early stages of the trial, the presence of live and viable mycobacteria: this is the basic condition that makes bacteria detection possible in BE, but it does not affect the performances of molecular analyses. This would also explain the similar median values for the detection period for both methods when assessing this group of samples. On the other hand, within naturally infected tissue, part of the bacterial population could have no longer been viable, making bacteriologic isolation less feasible but leaving the detection capability of PCR almost unchanged, which is the reason PCR was able to perform well for a longer period. Finally, as already mentioned, evaluations involving the fourth experimental batch confirmed the effect of freezing on keeping the infectious load active.

The choice to create both simple and multivariable regression models enabled us to notice some other interesting remarks: in fact, although only variations in the “method” and “contamination level” parameters, among those considered, seemed to significantly affect the probability of properly detecting the pathogen in meat or processed meat, our results suggest it is important to not overlook other variables potentially responsible for confounding effects. For instance, we noticed that, despite the lack of statistical significance of the results produced by comparing the two different procedures/sources of contamination, at first glance, an improvement seemed to occur in the performances for the analyses of the samples contaminated with infected lymph nodes compared with the other group. The extent of this improvement, however, was scaled down when we also considered other covariates, specifically when we also took into account the effects of a different bacterial count contaminating the matrix. In particular, for the microbiological examinations, the risk of not detecting the mycobacterium underwent a quite limited reduction (only 10%) when comparing results from testing the matrix in which naturally infected tissue was added to the results from the samples from the second and third batches. Nevertheless, this apparent “advantage” tends to be reversed if we analyze the data using a regression model also including the contamination level. Indeed, this latter model showed that the culturing method seemed to present a slightly higher risk of not detecting contamination in samples on the first batch compared with the other two batches (HR > 1). The worsening in analytical performances is probably due to the fact that the samples from the first group had a contamination level that negatively affected the BE output. Further proof of the reciprocal influence of the two variables considered was obtained by evaluating the calculated HR within the comparison of the groups with different contamination levels using a microbiological analysis. We can hypothesize that a more limited reduction in the risk when comparing a low-contamination batch and a high-contamination one against the reduction observed when comparing a low-contamination batch and a moderately contaminated one may be due to a confounding effect and to the co-occurring variation in both of the considered parameters in the latter paired comparison. Indeed, when we added the last covariate to the Cox model generated from the whole data set, the HR values for the effects of contamination level also showed a slight increase, pointing to a reduction in the differences between the groups. The same change was observed for the PCR-only data but was not present for the BE data, which instead presented an inversion of the hazard trends. This disturbance effect could underlie the above-mentioned unexpected result, considering that, unlike the second and third batches, the first and third batches differed in both contamination level and source, suffering from a further influence of this latter parameter in a different way for the two analytical techniques. For the sake of completeness, we report that the “method” parameter seemed to not suffer or provide any interference effect when introduced into the multivariable regression model.

From the results of this study, the role of meat in spreading infections with *M. bovis* to humans, despite being less relevant with respect to an inhalation route of infection, the ingestion of unpasteurized milk and milk products, and transmission by a dermal route, must be further and adequately evaluated in order to comprehensively quantify the real risk. To do this, we need to consider the many ways in which the meat is consumed. The chance to achieve heat inactivation of *M. bovis* in foodstuffs is correlated with the temperature/time ratio [34]. Moreover, it has been demonstrated that, under certain conditions, *M. bovis* could resist high temperatures and maintain its infectiveness and pathogenicity [35]. We estimate that the reduced temperature/time ratio to which fresh wild boar sausages are generally subjected during cooking could represent one of these conditions. In addition, we stress that the analytical detection of contamination, essential in prevention and monitoring activities, can be affected by many factors, which have to be taken into account. In more “natural” conditions, such as those simulated in the first experimental session, biomolecular analysis may represent a better choice compared with BE to identify a contaminated matrix, but even so, for the reasons reported above, expressing an unquestionable response about the potential of the food product as a means to transmit infection is not possible.

Data that suggest zoonotic tuberculosis is correlated with meat from wild species are still insufficient and, in any case, underestimated. The present study demonstrated the survival and viability of *M. bovis* in wild boar sausage artificially contaminated up to 23 days after preparation. Wild boar sausages are a type of foodstuff traditionally consumed as a fresh product within 2–3 days from manufacturing, after grilling or cooking using a plating process that, due to the reduced temperature/time ratio, is not always able to completely inactivate pathogenic microorganisms present within the food product. The remarkable increase in the number of animals killed during hunting activities, the treatment of meat from wild animals, from evisceration to storage procedures, as well as the proven presence of *M. bovis* in wild boars are all issues that need adequate hygienic safety assurance, specifically for the use of meat from wild fauna, in order to protect consumer health.

## Figures and Tables

**Table 1:** Order of markers used to identify the genetic profile of the *M. bovis* strain used in the experimental contamination phase of this work.

Markers
ETR-A
ETR-B
ETR-C
ETR-D
ETR-E
QUB 11a
QUB 11b
QUA 26
QUA 1895
QUB 15
VNTR 3232
MIRU 26

**Table 2:** Primers and probes used for real-time PCR [24].

Target	Primers/Probe	Concentration	Nucleotide Sequence (5′-3′)	Size (bp)
IS6110	EXT-1	10 µM	CCCGGACAGGCCGAGTTT	18
	INT-1	10 µM	CCCCATCGACCTACTACG	18
	5′-FAM/3′BHQ1	10 µM	AACTCAAGGAGCACATCAGCCG	22

**Table 3:** Results of the tests related to the first experimental batch.

Check	Time from Contamination	PCR Results	BE Results
1	T_0_ (0 days)	+	−
2	T_1_ (7 days)	+	−
3	T_2_ (15 days)	+	−
4	T_3_ (23 days)	+	+
5	T_4_ (33 days)	+	−
6	T_5_ (41 days)	+	−

**Table 4:** Results of the tests related to the second experimental batch.

Check	Time from Contamination	PCR Results	BE Results
1	T_0_ (0 days)	+	+
2	T_1_ (7 days)	+	+
3	T_2_ (15 days)	+	+
4	T_3_ (22 days)	+	+
5	T_4_ (30 days)	+	−
6	T_5_ (37 days)	+	−

**Table 5:** Results of the tests related to the third experimental batch.

Check	Time from Contamination	PCR Results	BE Results
1	T_0_ (0 days)	+	−
2	T_1_ (7 days)	+	−
3	T_2_ (14 days)	−	−
4	T_3_ (21 days)	−	−
5	T_4_ (32 days)	+	−
6	T_5_ (37 days)	−	−

**Table 6:** Results of the tests related to the fourth experimental batch.

Check	Time from Contamination	PCR Results	BE Results
1	T_0_ (0 days)	+	+
2	T_1_ (58 days)	+	+
3	T_2_ (120 days) *	=	=
4	T_3_ (182 days)	+	+
5	T_4_ (252 days)	+	+
6	T_5_ (342 days)	+	+

* Check not carried out due to a SARS-CoV2 emergency.

**Table 7:** Main results of statistical tests concerning the evaluated parameters and involving data from the whole dataset from the first, second and third experimental batches, and data separated according to the two different analytical techniques.

Categorical Variable	*p*-Value (Log-Rank Test)	Single Variable *p*-Value (Multivariable Model)	HR * (95% CI)	
			(Multivariable Model)	(Simple Regression Model)
Analytical method	0.021	0.039	0.308 (0.100–0.944) ^a^	0.308 (0.100–0.944) ^a^
Contamination level	0.026	0.045	0.459 (0.214–0.981) ^b^	0.434 (0.211–0.889) ^b^
Contamination level—BE	0.327	0.188	0.584 (0.262–1.302) ^b^	0.589 (0.277–1.253) ^b^
Contamination level—PCR	0.024	0.450	0.081 (0.000–55.250) ^b^	0.033 (0.000–6.494) ^b^
Contamination source	0.355	0.748	0.815 (0.235–2.828) ^c^	0.606 (0.195–1.887) ^c^
Contamination source—BE	0.866	0.942	1.048 (0.294–3.735) ^c^	0.906 (0.271–3.028) ^c^
Contamination source—PCR	0.141	0.721	0.083 (0.000–71525.211) ^c^	0.025 (0.000–132.032) ^c^

* The following categories were assumed to be the reference group for the HR calculation: ^(a)^ bacteriological cultural test (BE), ^(b)^ low contamination level, and ^(c)^ contamination through inoculation of the bacterial culture.

## Data Availability

Data is contained within the article.
